# High-Flow Nasal Cannula in Hypercapnic Respiratory Failure: A Systematic Review and Meta-Analysis

**DOI:** 10.1155/2020/7406457

**Published:** 2020-10-29

**Authors:** Yongkang Huang, Wei Lei, Wenyu Zhang, Jian-an Huang

**Affiliations:** The First Affiliated Hospital of Soochow University, Suzhou, China

## Abstract

**Background:**

Although the efficacy and safety of high-flow nasal cannula (HFNC) in hypoxemic respiratory failure are widely recognized, it is yet unclear whether HFNC can effectively reduce the intubation rate and mortality in hypercapnic respiratory failure. We performed a systematic review and meta-analysis to assess the safety and efficiency of HFNC in these patients.

**Methods:**

A systematic search of PubMed, Embase, and Cochrane Library (CENTRAL) was carried out. Two reviewers independently screened all references according to the inclusion criteria. We used the Cochrane risk-of-bias tool and the Newcastle–Ottawa Quality Assessment Scale to assess the quality of randomized controlled trials (RCTs) and cohort studies, respectively. Data from eligible trials were extracted for the meta-analysis.

**Results:**

Eight studies with a total of 621 participants were included (six RCTs and two cohort studies). Our analysis showed that HFNC is noninferior to noninvasive ventilation (NIV) with respect to intubation rate in both RCTs (OR = 0.92, 95% CI: 0.45–1.88) and cohort studies (OR = 0.94, 95% CI: 0.55–1.62). Similarly, the analysis of cohort studies showed no difference in reducing mortality rates (OR = 0.96, 95% CI: 0.42–2.20). Based on RCTs, NIV seemed more effective in reducing mortality (OR = 1.33, 95% CI: 0.68–2.60), but the intertreatment difference was not statistically signiﬁcant. Furthermore, no significant differences were found between HFNC and NIV relating to change of blood gas analysis or respiratory rate (MD = −0.75, 95% CI: −2.6 to 1.09). Likewise, no significant intergroup differences were found with regard to intensive care unit stay (SMD = −0.07, 95% CI: 0.26 to 0.11). Due to a physiological friendly interface and variation, HFNC showed a significant advantage over NIV in patients' comfort and complication of therapy.

**Conclusion:**

Despite the limitations noted, HFNC may be an effective and safe alternative to prevent endotracheal intubation and mortality when NIV is unsuitable in mild-to-moderate hypercapnia. Further high-quality studies are needed to validate these findings.

## 1. Introduction

Respiratory failure, which can occur due to several different diseases and conditions, is a common syndrome occurring in the intensive care unit (ICU) [1]. Endotracheal intubation is usually performed only when the patient is deteriorating despite optimal drug and common oxygen therapy, and it often results in extra medical expenses, longer hospital stay, and even higher mortality [2, 3]. Thus, it is crucial to protect patients from acute respiratory failure and avoid, as far as possible, invasive mechanical ventilation.

Noninvasive ventilation (NIV) is recommended by guidelines to avoid intubation and improve outcomes [4]. However, many patients who need respiratory support may be excluded by the technicians for comorbidities such as emphysema and oversecretion of sputum [5, 6]. Besides the contraindications, higher expenditure and numerous potential adverse events are presented during NIV, such as skin damage, eye irritation, interface intolerance, diet, and sputum retention, which cause discomfort and may lead to the termination of NIV to some extent [7].

High-flow nasal cannula (HFNC) is a simple system composed of an air-oxygen blender, active heated humidifier, single heated circuit, and nasal cannula and can deliver high-rate humidified oxygen (up to 60 L/min) through a nasal cannula. It has been deemed an effective and less costly alternative among children to alleviate respiratory distress and prevent extubation failure [8, 9]. In recent years, HFNC has become increasingly popular in the treatment of respiratory failure in adults [10]. HFNC is reportedly superior to conventional oxygen therapy and can be as effective as NIV in patients with acute hypoxemic respiratory failure [11]. However, it is still unclear whether HFNC is an effective tool to reduce the intubation rate and mortality in patients suffering from hypercapnic respiratory failure.

## 2. Methods

This systematic review and meta-analysis was registered at PROSPERO (http://www.crd.york.ac.uk/prospero; CRD: 42020173744) and designed as per the Cochrane Handbook for Systematic Reviews of Interventions [12] and reported according to the PRISMA guidelines.

### 2.1. Literature Searching Strategy

We performed a comprehensive search of electronic databases including PubMed, Embase, and Cochrane Central Register of Controlled Trials (CENTRAL) up to May 2020, using the keywords and their synonyms, and the search was updated on September 12, 2020. Terms were related to the intervention and modified according to each database's index term, such as Medical Subject Heading (MeSH) and Emtree. No language restrictions or publication year were applied when searching PubMed and CENTRAL, while it was limited to clinical studies on humans on Embase. Relevant citations from the references listed in each identified study were also taken into consideration for eligibility. Detailed search terms are shown in Appendix 1 (Supplementary Materials (available here)).

### 2.2. Inclusion and Exclusion Criteria

Studies in our review had to meet all of the following criteria:

1. Type of participants: participants must be adults (age >16 years) with acute hypercapnic respiratory failure (PaCO_2_ > 45 mmHg)
2. Type of intervention and comparator: comparing HFNC with NIV
3. Type of studies: a randomized controlled trial (RCT) or a cohort study
4. Containing any one of the following outcomes: intubation rate; mortality; blood gas analysis (arterial partial pressure of oxygen (PaO_2_), arterial partial pressure of carbon dioxide (PaCO_2_), and pH); respiratory rate, patient comfort; and complication of the therapy

Exclusion criteria:

1. Studies with the same data or overlapping data by the same authors
2. Studies without any one of the predetermined outcomes

### 2.3. Quality Assessment

The quality of all selected studies was assessed independently by two reviewers (HYK and ZWY). RCTs were evaluated according to the Cochrane risk-of-bias tool which includes the following items: random sequence generation, allocation concealment, blinding of participants and personnel, blinding of outcome assessment, incomplete outcome data, selective reporting, and other biases. On the contrary, a cohort study was graded according to the Newcastle–Ottawa Quality Assessment Scale [13] with regard to selection, comparability, and outcome.

### 2.4. Data Extraction

Two researchers (HYK and ZWY) independently extracted the following data from each study: characters of the study (first author, publication year, country, number of participants, case source, cause of hypercapnic respiratory failure, and major inclusion criteria); characters of the participants (demographic variation and basic blood gas analysis); and the primary and secondary outcomes. Any disagreements between the two authors were resolved by consensus or cross-checking with a third author (LW). Additional information was collected through communication with the principal investigator by email if necessary.

### 2.5. Data Analysis

Statistical analysis was performed by an independent researcher adept in statistics using Cochrane systematic review software Review Manager (RevMan; version 5.4.0; The Nordic Cochrane Centre, The Cochrane Collaboration, Copenhagen, 2014). Continuous variables were reported as mean and standard derivation (SD), while dichotomous variables were shown as frequency or proportion. The results were displayed in forest plots. An initial test for clinical, methodological, and statistical heterogeneities was conducted, and we used the chi-square test with *P* < 0.1 and *I*^2^ > 50% to indicate statistical significance. Random-effect model was applied in the presence of statistical heterogeneity; otherwise, the fixed-effect model was chosen. For continuous data, we calculated the mean difference (MD) or standard mean difference (MD) and 95% confidence interval (CI), and for dichotomous data, we calculated the odds ratio (OR) and 95% CI.

## 3. Results

### 3.1. Study Selection and Characteristics

**Figure 1:**
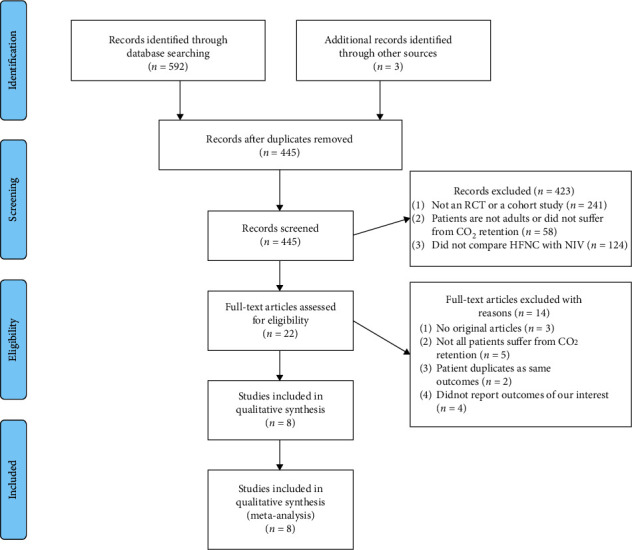
Study flow.

In all, 595 citations were retrieved through literature search. We excluded 150 duplicates identified by the title and authors and omitted 423 studies that did not fulfill the inclusion criteria. We tried to obtain the full text of the remaining 22 searches. Finally, eight studies, comprising six parallel RCTs [14–19] and two cohort studies [20, 21], involving 621 participants were enrolled in the analysis. The flowchart of the study is shown in Figure 1.

Tan et al. conducted their study in two large tertiary care hospitals, while the remaining studies included were all carried out in a single center. Seven studies were performed in Asia and one in Greece. Most patients were admitted to the ICU or respiratory ICU. Three of six RCTs recruited only extubated patients, while patients with acute-exacerbation chronic obstructive pulmonary disease (AECOPD) were the main cause of hypercapnic respiratory failure in another two RCTs and both cohort studies. One study did not mention the cause of admittance. Characteristics of the participants and studies are shown in Tables 1–3, respectively.

### 3.2. Risk of Bias within Studies

**Figure 2:**
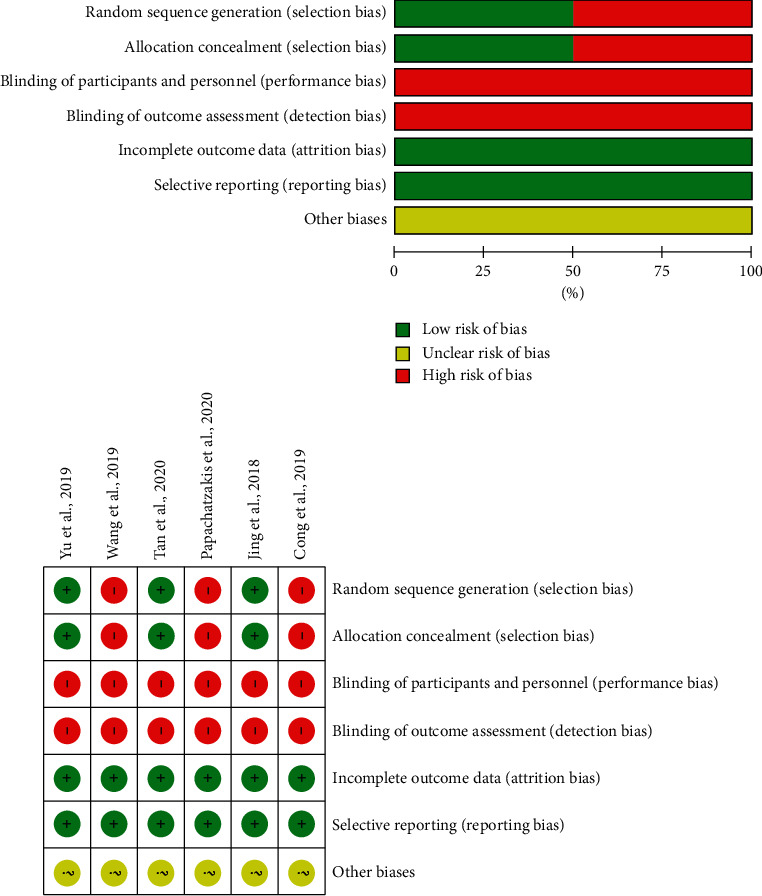
Quality assessment of each eligible trial.

Three studies [15, 16, 18] did not detail the method of their random sequence generation and allocation concealment, which may cause selection bias. Blinding of participants was not performed in all RCTs owing to different appearances of the devices. There was no bias in detection, attrition, and reporting. The quality assessment of each eligible trial is shown in Figure 2.

### 3.3. Clinical Effectiveness

#### 3.3.1. Effect on Intubation Rate and Mortality

**Figure 3:**
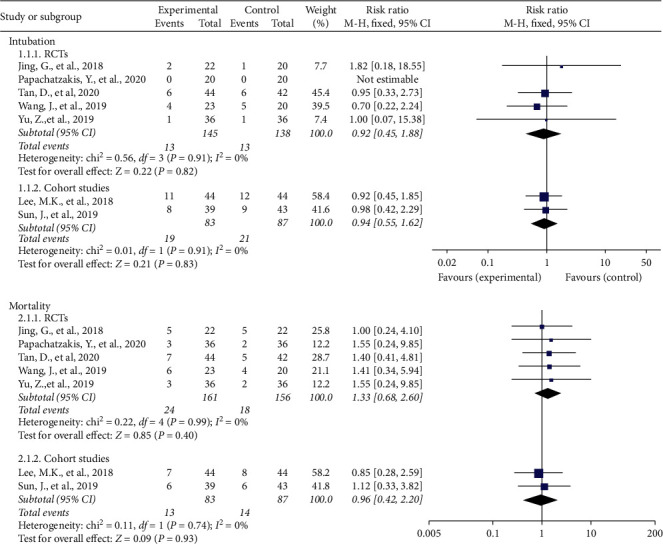
Intubation and mortality.

Both cohort studies and five RCTs that reported intubation and mortality were considered in our meta-analysis. The pooled data showed that HFNC was noninferior to NIV in preventing intubation or reintubation both in RCTs (OR = 0.92, 95% CI: 0.45–1.88) and cohort studies (OR = 0.94, 95% CI: 0.55–1.62). Similarly, the synthesis of cohort studies (OR = 0.96, 95% CI: 0.42–2.20) in reducing mortality indicates no difference. NIV seems to be more effective in reducing mortality in RCTs (OR = 1.33, 95% CI: 0.68–2.60), but the between-treatment difference was not statistically signiﬁcant. Forest plot of intubation and mortality is shown in Figure 3.

#### 3.3.2. Effect on Blood Gas Analysis and Respiratory Rate

Of all eligible studies, seven reported at least one of the following blood gas analysis outcomes including PaO_2_, PaCO_2_, and pH. The variables at 12 h or 24 h after initiation of therapy were collected and merged.

**Figure 4:**
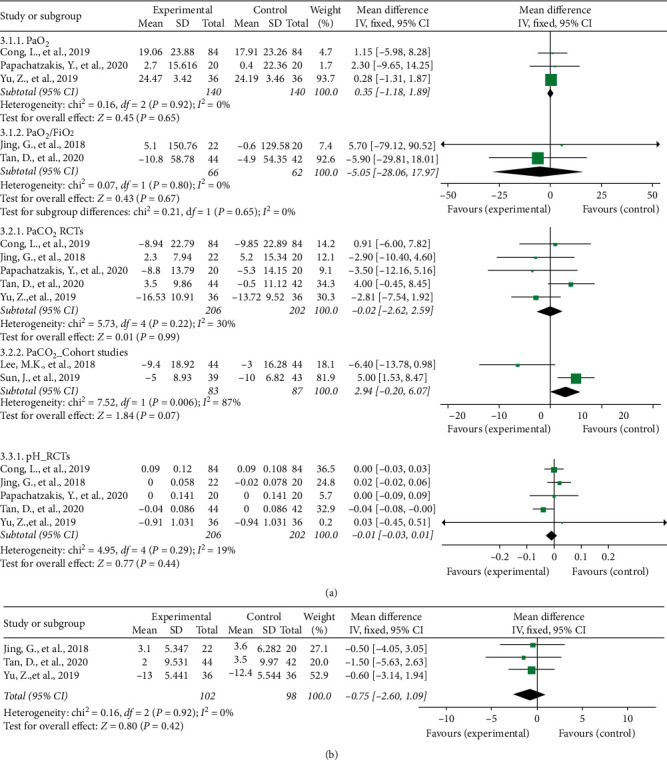
(a) Blood gas analysis. (b) Respiratory rate.

There was no difference between HFNC and NIV in oxygenation improvement (MD = 0.35, 95% CI: −1.18 to 1.89 in PaO_2_ and MD = −5.05 95% CI: −28.06 to 17.97 in PaO_2_/FiO_2_); removing carbon dioxide (MD = −0.02, 95% CI: −2.62 to 2.59 for RCTs; MD = 2.94, 95% CI: −0.20 to 6.07 for cohort studies); pH change (MD = −0.01, 95% CI: −0.03 to 0.01); or alleviating respiratory distress (MD = −0.75, 95% CI −2.6 to –1.09). Likewise, two cohort studies showed a similar tendency without a significant difference. Forest plots of blood gas analysis and respiratory rate are shown in Figure 4.

#### 3.3.3. Effect on ICU Stay

**Figure 5:**
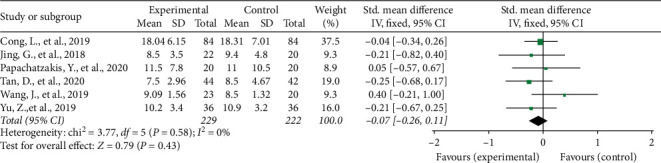
ICU stay.

All six RCTs reported the patients' stay in the ICU and were pooled into the analysis. The results suggested that both therapies were similar with respect to ICU stay (SMD = −0.07, 95% CI: −0.26 to 0.11). Forest plot of ICU stay is shown in Figure 5.

#### 3.3.4. Patients' Comfort and Complications of Therapy

Three studies [14, 15, 19] that reported patients' comfort indicated a statistically significant effect in support of HFNC. Similarly, four studies [16, 17, 19, 20] that reported complications of therapy showed a significantly lower ﬂatulence rate and incidence of nasal-facial breakdown rate in the HFNC group than in the NIV group (all *P* < 0.05). Besides, fewer patients in the HFNC group needed less airway care intervention. The summary of patients' comfort and complication is shown in Table 4.

#### 3.3.5. Heterogeneity Analysis

Significant heterogeneity was tested in PaCO_2_ of the cohort studies (*I*^2^ = 87%, *c*^2^ = 7.52, *P*=0.006), but not among the RCTs (*I*^2^ = 30%, *χ*^2^ = 5.73, *P*=0.22). There were good consistency and low heterogeneity among the other variables analyzed.

## 4. Discussion

The efficacy and safety of high-flow nasal cannula (HFNC) in patients with hypercapnic respiratory failure have been debatable in recent years. Pisani et al. [22] reported that COPD patients recovering from an acute exacerbation and with persistent hypercapnia showed a statistically significant response in terms of PaCO_2_ decrease. This was strengthened by another retrospective study among hypercapnic AECOPD patients that reported a significant treatment effect in removing CO_2_, especially for acidotic patients (baseline pH < 7.35) [23]. It was also reported that the use of HFNC resulted in dyspnea relief and hypercapnia improvement [24] and could effectively mitigate diaphragm fatigue [25]. In contrast, HFNC did not decrease PaCO_2_ in the hypercapnic subgroup of another cross-sectional study [26] and patients with mild-to-moderate AECOPD in the trial of Yang et al. [25].

In this meta-analysis, we found that HFNC is as effective as NIV in preventing endotracheal intubation and mortality. We also found that HFNC has a similar effect as NIV on pH change, improving oxygenation, removing carbon dioxide, alleviating respiratory distress, and ICU stay. This could be attributed to the following factors. First, HFNC decrease in the dead space of the air channel improves alveolar ventilation and washes out carbon dioxide in the anatomical dead space, thereby leading to effective PaCO_2_ reduction [27–29]. Second, HFNC decreased inhalation-expiration ratio and respiratory rate, improved breathing patterns, and subsequently reduced the work of breath [30–32]. Di Mussi et al. [33] stated that HFNC after extubation significantly decreased the neuroventilatory drive and work of breathing compared with conventional oxygen therapy. Third, by delivering high-flow gas, HFNC may produce a flow and leakage-dependent positive end-expiratory pressure (PEEP) and prevent airway collapse that may be beneficial for alveolar recruitment on the one hand and improve the mismatch between ventilation and perfusion on the other hand [34–36]. Besides, the physiological friendly gas perks up mucosal function and facilitates secretion clearance. All the factors may mitigate lung injury and induce a noninferior outcome. Given the friendly interface that would not disturb speaking, spitting, or eating and a stable flow with warm and well-humidified gas, HFNC was undoubtedly superior to NIV with regard to patients' comfort and therapy-related complications.

Our study has several limitations. First is the fact that eligible clinical studies on the use of HFNC in hypercapnic respiratory failure were limited. Second is the methodological issues of the included studies, for example, explicit randomization in partial studies, lack of blinding for all RCTs, and single-center design of most studies in Asia. These factors may lead to bias and weaken the strength of evidence. Besides, there was significant heterogeneity in the analysis of PaCO_2_ in cohort studies (*I*^2^ = 87%, *χ*^2^ = 7.52, *P*=0.006), which was speculated to be caused by differences in the initial flow rate of HFNC and the overall severity of patients, deduced on the basis of different places from where patients were recruited. Thus, the quality of the included studies ranged from moderate to low.

## 5. Conclusion

Despite the limitations noted, HFNC may be an effective and safe alternative to prevent endotracheal intubation and mortality when NIV cannot be performed in mild-to-moderate cases of hypercapnia. Large and well-structured trials are needed to validate these findings.

## Figures and Tables

**Table 1:** Characteristics of cohort studies.

Authors, year	Participates of the cohort (HFNC/NIV)	Case source	Cause of hypercapnic respiratory failure	Follow-up (days)	Location	Major inclusive criteria	Initial indications of HFNC	Initial indications of NIV	Primary outcome	NOS scores
Sun et al., 2019	39/43	ICU	AECOPD or pulmonary infection with COPD	28	China	COPD or acute respiratory failure by a secondary diagnosis of COPD with a respiratory acidosis (pH ≤ 7.35 and P_a_CO_2_ ≥ 50 mmHg)	Initial FiO_2_ in the HFNC group was 0.3 (0.2–0.4), and the gas flow rate was 50 L/min (40–50).	Initial FiO_2_ in the NIV group was 0.4 (0.3–0.6), inspiratory airway pressure was 10 cm H_2_O (8–12), and expiratory airway pressure was 4 cm H_2_O (4-5). Mean expiratory tidal volume during the first 24 hrs of NIV treatment was 5.4 ± 2.4 ml/kg of predicted body weight.	Treatment failure and 28-day mortality	5

Lee et al., 2018	44/44	Respiratory ward	AECOPD	30	South Korea	AECOPD with moderate hypercapnic acute respiratory failure ((PaO_2_)/FiO_2_ < 200 mmHg, PaCO_2_ > 45 mmHg, and 7.25 < pH < 7.35 on room air)	Beginning with FiO_2_ > 50% and a flow of 35 L/min and then titrating flow to 45–60 L/min if tolerated. FiO_2_ was subsequently adjusted to maintain an oxygen saturation of 92% or more.	The expiratory pressure was set at 5 cm H_2_O pressure, and inspiratory pressure was initially set at 10 cm H_2_O and then increased in increments of 2–4 to 20 cm H_2_O or the maximum tolerated over 1 hour. The BiPAP level was adjusted to maintain an oxygen saturation of 92% or more.	Intubation rate and 30-day mortality	6

**Table 2:** Characteristics of the RCTs.

Authors, year	Case source	Subjects	Major inclusive criteria	Follow-up (days)	Location	Outcome	Indications of HFNC	Indications of NIV	Trial registration number
Jing et al., 2018	—	42	Patients with hypercapnia (PaCO_2_ > 45 mmHg) at the time of extubation and met the “pulmonary infection control window” criteria	28	China	The primary outcome parameters were ABG analysis and vital signs. Secondary outcomes included duration of respiratory support, length of ICU stay, the patients' comfort score, and incidence of adverse events.	The humidifier temperature was set at 37°C, and the fraction of inspired oxygen was adjusted to maintain oxygen saturation recorded by pulse oximetry (SpO_2_) at 88–92%.	The IPAP was initiated at 10–12 cm H_2_O, EPAP started at 4-5 cm H_2_O, and subsequent adjustments were based on the patients' ABGs.	ClinicalTrials (NCT03458364)

Yu et al., 2019	ICU	72	Extubation patients with hypercapnia (PaCO_2_ > 50 mmHg)	Not mentioned	China	The ABG, respiratory rate, heart rate, mean arterial pressure, reintubation rate, mortality, intensive care unit stay, and incidence of adverse events.	The humidifier temperature was set at 37°C, and the fraction of inspired oxygen was adjusted according to ABGs and patients' symptoms and signs.	The IPAP was initiated at 10～14 cm H_2_O, and EPAP was 4～6 cm H_2_O. Variables were adjusted according to ABGs and patients' symptoms and signs.	No

Wang et al., 2019	RICU	43	AECOPD patients with hypercapnic respiratory failure	28	China	The treatment failure rate, tracheal intubation rate, complications, and 28-day survival rate.	Both the flow rate and the fraction of inspired oxygen were according to ABGs and patients' symptoms and signs.	Both IPAP and EPAP were adjusted according to ABGs and patients' symptoms and signs.	No

Cong et al., 2019	ICU	168	AECOPD patients	Not mentioned	China	The primary endpoint was ABG analysis. Secondary clinical endpoints included ventilation support time, hospitalization days and complications, comfort, and nursing satisfaction.	The air temperature was set at 37°C at a flow rate of 30–35 L/min.	The IPAP was set at 10 cm H_2_O, and EPAP was set at 5 cm H_2_O at the beginning and gradually increased after the patient adapted. Patients' symptoms and signs were monitored, and FiO_2_ was adjusted to ensure oxygen saturation2.	No
Tan et al, 2020	ICU	86	Extubation patients with COPD patients with hypercapnic respiratory failure	28	China	The primary endpoint was treatment failure. Secondary outcomes included arterial blood gas analysis and vital signs.	The initial airflow was set at 50 L/min and adjusted according to patient tolerance. The HFNC was set to an absolute humidity of 44 mg H_2_O/L, temperature was set to 37°C, and FiO_2_ was adjusted to maintain an SpO_2_ of 88–92%.	The initial EPAP was set to 4 cm H_2_O, while the IPAP was initially set to 8 cm H_2_O. The pressure level was gradually increased to achieve a satisfactory tidal volume with acceptable tolerance. The pressure level and the fraction of inspiration oxygen (FiO_2_) were adjusted to maintain 88–92% SpO_2_.	Chictr.org (ChiCTR1800018530)

Papachatzakis et al., 2020	ED	40	Patients suffering acute respiratory failure type 2	Not mentioned	Greece	Endpoints were intubation and mortality rate, length of hospitalization, duration of therapy, and possible differences between vital signs, ABGs, and comfort.	The initial airflow was set at a flow of 35 L/min, titrating flow upward if tolerated to 45–50 L/min, in order to maintain SaO_2_ > 90% or according to specific clinical orders.	Expiratory and inspiratory pressures were gradually increased to the maximum tolerated over 1 h, in order to maintain SaO_2_ > 90%, or according to specific clinical orders.	No

ED: emergency department; IPAP: inspiratory positive airway pressure; EPAP: expiratory pressure airway pressure; ABG: arterial blood gases.

**Table 3:** Characteristics of the participants.

Authors, year	Age (years)		Gender (male/total)		APACHE II score			Respiratory rates (times/minute)		pH		PaO_2_ (mmHg) or PaO_2_/FiO_2_*∗*(mmHg)		PaCO_2_ (mmHg)		Respiratory support duration (days or hours*∗*)	
	HFNC	NIV	HFNC	NIV	HFNC		NIV	HFNC	NIV	HFNC	NIV	HFNC	NIV	HFNC	NIV	HFNC	NIV
RCTs																	
Jing et al., 2018	77.4 ± 6.8	73.9 ± 6.9	?/22	?/20	11.8 ± 3.1	10.42.5		18.3 ± 3.5	19.2 ± 4.1	7.46 ± 0.04	7.44 ± 0.06	235.8 ± 77.0*∗*	250.8 ± 75.8*∗*	52.4 ± 6.4	53.7 ± 8.6	2.73 ± 1.95	4.07 ± 4.40
Yu et al., 2019	62.4 ± 10.1	63.5 ± 11.2	24/36	21/36	28.6 ± 2.8	28.5 ± 3.4		32 ± 4.4	33 ± 4.3	7.26 ± 0.03	7.26 ± 0.03	56.84 ± 2.77	56.92 ± 2.89	73.56 ± 6.9	73.5 ± 6.23	—	—
Wang et al., 2019	71.26 ± 7.39	72.85 ± 6.65	13/23	12/20	18.35 ± 2.19	18.9 ± 2.59		30.91 ± 2.13	30.35 ± 2.68	7.23 ± 0.19	7.24 ± 0.02	57.17 ± 5.68	59.55 ± 6.48	67.13 ± 4.25	66.05 ± 3.03	7.96 ± 1.72	6.8 ± 1.26
Cong et al., 2019	66.91 ± 7.38	67.88 ± 8.38	48/84	50/48	—	—		—	—	7.25 ± 0.08	7.27 ± 0.09	53.10 ± 16.22	54.08 ± 15.33	72.11 ± 16.31	72.91 ± 16.41	10.02 ± 5.11	9.55 ± 4.78
Tan et al, 2020	68.4 ± 9.3	71.4 ± 7.8	27/44	23/42	14 (11–18.8)	13 (10.8–16)		18 (16–23)	21 (16–26)	7.48 (7.42–7.51)	7.45 (7.40–7.49)	239.2 ± 47.0*∗*	229.3 ± 42.0*∗*	50.5 (48–57.8)	53 (48.8–61.3)	83.9 ± 33.1*∗*	70.9 ± 30.6*∗*
Papachatzakis et al., 2020	76 ± 13.4	78.1 ± 8.1	10/20	9/20	21.6 ± 8.9	19.6 ± 6.1		—	—	7.1 ± 0.1	7.1 ± 0.1	76.4 ± 28.9	65.2 ± 12.9	60.4 ± 9.9	62.1 ± 10.3	2 ± 1	2 ± 9
Cohort studies																	

Lee et al., 2018	73 (68–79)	77 (71–70)	28/44	29/44	—		—	24 (20–28)	24 (22–29)	7.32 ± 0.28	7.31 ± 0.29	134.8 ± 7.3*∗*	134.5 ± 7.5*∗*	56.4 ± 10.1	52.6 ± 8.8	7 (5–10)	8 (6–10)
Sun et al., 2019	73.2 ± 9.0	70.4 ± 7.4	24/39	30/43	18.4 ± 2.7		17.3 ± 3.4	28.1 ± 3.3	27.0 ± 3.5	7.31 (7.29–7.33)	7.30 (7.28–7.32)	138.2 ± 6.6*∗*	140 ± 6.6*∗*	56 (53–62)	59 (55–62)	5 (4–7)	6 (5–8)

^*∗*^distinguishes the two indicators of the variable. For example, “day” was chosen by Jing et al. (the first RCT in the table) in their study to measure the respiratory support duration, while Tan et al. (the fifth RCT in the table) prefer “hours” to be the measurement.

**Table 4:** Summary of patients' comfort and complication.

	Nasofacial skin breakdown	Gastric and intestinal flatulence	Comfort scores	Airway care interventions
Sun et al., 2019	5.1% vs. 20.9%, *P* < 0.05	—	—	5 (4–7) vs. 8 (7–10), *P* < 0.001
Jing et al., 2018	—	—	3.55 ± 2.01 vs. 5.15 ± 2.28, *P*=0.02	9.09% vs. 45%, *P*=0.03
Cong et al., 2019	—	—	75% vs. 57%, *P*=0.008	—
Yu et al., 2019	—	5.6% vs. 25%, *P*=0.022	—	—
Wang et al., 2019	8.7% vs. 40%, *P*=0.028	13.0% vs. 45.0%, *P*=0.039	—	—
Tan et al., 2020	0 vs. 9.6%, *P*=0.027	—	7 (6–8) vs. 5 (4–7), *P* < 0.001	6 (4–7) vs. 7 (5–9.3), *P*=0.006

## Data Availability

The studies included can be found on PubMed except two RCTs, of which one was indexed in Embase (Yu ZH et al., Academic Journal of Second Military Medical University 2019, https://doi.org/10.16781/j.0258-879x.2019.09.0989), and the other (Wang et al., Chin J Mod Med 2019, https://kns.cnki.net/kcms/detail/43.1225.R.20190613.1310.010.html) could be found on Chinese National Knowledge Infrastructure.

## Supplementary Materials

Supplementary Materials This section includes Appendix 1 with detailed search terms.
