# Supplementary material for: High-Flow Nasal Cannula in Hypercapnic Respiratory Failure: A Systematic Review and Meta-Analysis
Source: Can Respir J. 2020 Oct 29;2020:7406457. doi: 10.1155/2020/7406457 (PMC7647788; doi:10.1155/2020/7406457)
Supplement: Supplementary Materials — This section includes Appendix 1 with detailed search terms. [file 7406457.f1.zip › 7406457.f1/Search_Strategy_Cochrane.pdf]

Search Name: HFNC
Date Run: 12/09/2020 09:26:10
Comment: HFNC vs NIV

ID	Search	Hits
#1	MeSH descriptor: [Pulmonary Disease, Chronic Obstructive] explode all trees	5683
#2	(COAD):ti,ab,kw	81
#3	(COPD):ti,ab,kw	15975
#4	(Chronic Obstructive Airway Disease):ti,ab,kw	1613
#5	(Chronic Airflow Obstructions):ti,ab,kw	2
#6	(Chronic Airflow Obstruction):ti,ab,kw	574
#7	(Chronic Obstructive Pulmonary Disease):ti,ab,kw	13198
#8	#1 or #2 or #3 or #4 or #5 or #6 or #7	20083
#9	(hypercarbia):ti,ab,kw	150
#10	MeSH descriptor: [Hypercapnia] explode all trees	482
#11	(CO2 retention):ti,ab,kw	128
#12	(Hypercapnia):ti,ab,kw	1319
#13	#10 or #11 or #12	1413
#14	#8 or #13	21153
#15	(high-flow oxygen therapy):ti,ab,kw	715
#16	(HFNC):ti,ab,kw	392
#17	(high-flow nasal cannula):ti,ab,kw	751
#18	(nasal high-flow oxygen therapy):ti,ab,kw	576
#19	(nasal High-flow):ti,ab,kw	1085
#20	(High-Velocity Nasal Insufflation):ti,ab,kw	8
#21	#15 or #16 or #17 or #18 or #19 or #20	1252
#22	#14 and #21	220
