# Supplementary material for: High-Flow Nasal Cannula in Hypercapnic Respiratory Failure: A Systematic Review and Meta-Analysis
Source: Can Respir J. 2020 Oct 29;2020:7406457. doi: 10.1155/2020/7406457 (PMC7647788; doi:10.1155/2020/7406457)
Supplement: Supplementary Materials — This section includes Appendix 1 with detailed search terms. [file 7406457.f1.zip › 7406457.f1/Search_Strategy_Embase.pdf]

Embase®

Embase Session Results

No.	Query	Results
**#24**	**#15** AND **#21** AND [humans]/lim AND [clinical study]/lim	188
**#23**	**#15** AND **#21** AND [humans]/lim	305
**#22**	**#15** AND **#21**	315
**#21**	**#16** OR **#17** OR **#18** OR **#19** OR **#20**	2,453
**#20**	**'nasal high-flow oxygen therapy'**:ab,kw,ti	48
**#19**	**'high-flow oxygen therapy'**:ab,kw,ti	347
**#18**	**'high flow nasal cannula'**:ab,kw,ti	1,855
**#17**	**'high flow nasal cannula'**/exp OR **'high flow nasal cannula oxygen therapy'**/exp OR **'high flow nasal cannula therapy'**/exp OR **'high flow nasal cannula oxygen'**/exp	552
**#16**	**'hfnc'**:ab,kw,ti	1,089
**#15**	**#9** OR **#14**	182,767
**#14**	**#10** OR **#11** OR **#12** OR **#13**	23,805
**#13**	**'co2 retention'**:kw,ti,ab	167
**#12**	**'hypercapnia'**:kw,ti,ab	14,215
**#11**	**'hypercapnia'**:kw,ti,ab	14,215
**#10**	**'hypercapnia'**/exp	20,049
**#9**	**#1** OR **#2** OR **#3** OR **#4** OR **#5** OR **#6** OR **#7** OR **#8**	161,973
**#8**	**'chronic airflow obstruction'**:ti,kw,ab	704
**#7**	**'chronic airflow obstructions'**:ti,kw,ab	3
**#6**	**'chronic obstructive lung disease'**:ti,kw,ab	6,168
**#5**	**'chronic obstructive airway disease'**:ti,kw,ab	499
**#4**	**'coad'**:ti,kw,ab	549
**#3**	**'chronic obstructive pulmonary disease'**:ti,ab,kw	72,510
**#2**	**'copd'**:ti,ab,kw	90,435
**#1**	**'chronic obstructive lung disease'**/exp OR **'chronic obstructive lung disease'**	137,826

ELSEVIER

© 2020 RELX Intellectual Properties SA. All rights reserved.
Embase, RELX Group and the RE symbol are trade marks of RELX Intellectual Properties SA, used under license.
