# Supplementary material for: High-Flow Nasal Cannula in Hypercapnic Respiratory Failure: A Systematic Review and Meta-Analysis
Source: Can Respir J. 2020 Oct 29;2020:7406457. doi: 10.1155/2020/7406457 (PMC7647788; doi:10.1155/2020/7406457)
Supplement: Supplementary Materials — This section includes Appendix 1 with detailed search terms. [file 7406457.f1.zip › 7406457.f1/Search_Strategy_PubMed.pdf]

Search number	Query
5	("HFNC" OR "high-flow nasal cannula" OR "high-flow oxygen therapy" OR "nasal high-flow oxygen therapy" OR "Nasal High-flow" OR "nasal high-flow" OR "High-Velocity Nasal Insufflation" OR "high flow oxygen" OR "long-term humidification") AND (("Pulmonary Disease, Chronic Obstructive"[Mesh] OR "COPD" OR "Chronic Obstructive Pulmonary Disease" OR "COAD" OR "Chronic Obstructive Airway Disease" OR "Chronic Obstructive Lung Disease" OR "Chronic Airflow Obstructions" OR "Chronic Airflow Obstruction") OR ("hypercapnia"[MeSH Terms] OR (((hypercapnia) OR hypercarbia) OR CO2 retention)))
4	("Pulmonary Disease, Chronic Obstructive"[Mesh] OR "COPD" OR "Chronic Obstructive Pulmonary Disease" OR "COAD" OR "Chronic Obstructive Airway Disease" OR "Chronic Obstructive Lung Disease" OR "Chronic Airflow Obstructions" OR "Chronic Airflow Obstruction") OR ("hypercapnia"[MeSH Terms] OR (((hypercapnia) OR hypercarbia) OR CO2 retention))
3	"hypercapnia"[MeSH Terms] OR (((hypercapnia) OR hypercarbia) OR CO2 retention)
2	"Pulmonary Disease, Chronic Obstructive"[Mesh] OR "COPD" OR "Chronic Obstructive Pulmonary Disease" OR "COAD" OR "Chronic Obstructive Airway Disease" OR "Chronic Obstructive Lung Disease" OR "Chronic Airflow Obstructions" OR "Chronic Airflow Obstruction"
1	"HFNC" OR "high-flow nasal cannula" OR "high-flow oxygen therapy" OR "nasal high-flow oxygen therapy" OR "Nasal High-flow" OR "nasal high-flow" OR "High-Velocity Nasal Insufflation" OR "high flow oxygen" OR "long-term humidification"
